# Multiple criteria decision analysis in the context of health technology assessment: a simulation exercise on metastatic colorectal cancer with multiple stakeholders in the English setting

**DOI:** 10.1186/s12911-017-0524-3

**Published:** 2017-10-26

**Authors:** Aris Angelis, Gilberto Montibeller, Daniel Hochhauser, Panos Kanavos

**Affiliations:** 1 Department of Health Policy and Medical Technology Research Group, LSE Health, London School of Economics and Political Science, London, UK; 2 School of Business and Economics, Management Science and Operations Group, Loughborough University, London, UK; 3 UCL Cancer Institute, London, UK

**Keywords:** Multiple criteria decision analysis (MCDA), Health technology assessment (HTA), Advance Value Framework (AVF), Metastatic colorectal cancer (mCRC), England, National Institute for Health and Care Excellence (NICE), Value assessment, New medicines

## Abstract

**Background:**

Multiple criteria decision analysis (MCDA) has appeared as a methodology to address limitations of economic evaluation in health technology assessment (HTA), however there are limited empirical evidence from real world applications. The aim of this study is to test in practice a recently developed MCDA methodological framework known as Advance Value Framework (AVF) through a proof-of-concept case study engaging multiple stakeholders.

**Methods:**

A multi-attribute value theory methodological process was adopted involving problem structuring, model building, model assessment and model appraisal phases. A facilitated decision analysis modelling approach was used as part of a decision conference with thirteen participants. An expanded scope of the National Institute for Health and Care Excellence (NICE) remit acted as the study setting with the use of supplementary value concerns. Second-line biological treatments were evaluated for metastatic colorectal cancer (mCRC) patients having received prior chemotherapy, including cetuximab monotherapy, panitumumab monotherapy and aflibercept in combination with FOLFIRI chemotherapy. Initially 18 criteria attributes were considered spanning four value domains relating to therapeutic impact, safety profile, innovation level and socioeconomic impact.

**Results:**

Nine criteria attributes were finally included. Cetuximab scored the highest overall weighted preference value score of 45.7 out of 100, followed by panitumumab with 42.3, and aflibercept plus FOLFIRI with 14.4. The relative weights of the two most important criteria (overall survival and Grade 4 adverse events) added up to more than the relative weight of all other criteria together (52.1%). Main methodological limitation was the lack of comparative clinical effects across treatments and challenges included the selection of “lower” and “higher” reference levels on criteria attributes, eliciting preferences across attributes where participants had less experience, and ensuring that all attributes possess the right decision theory properties.

**Conclusions:**

This first application of AVF produced transparent rankings for three mCRC treatments based on their value, by assessing an explicit set of evaluation criteria while allowing for the elicitation and construction of participants’ value preferences and their trade-offs. It proved it can aid the evaluation process and value communication of the alternative treatments for the group participants. Further research is needed to optimise its use as part of policy-making.

## Background

The assessment and appraisal of new and expensive medicines by health technology assessment (HTA) bodies, health insurers, and gatekeeper agencies has received considerable attention in recent years, especially in countries with publicly funded health care systems. This is a consequence of negative, and sometimes controversial, recommendations on the funding of new medicines due to their high costs. In several cases these medicines relate to treatments for severe diseases with high burden, leading to high patient dissatisfaction and public criticism.

As a result, the methodological aspects for assessing and appraising new medicines have been placed under scrutiny. The use of QALYs (quality adjusted life years) as part of economic evaluations in HTA, although it is a reasonable measure of health gain, it has been argued as inadequate to express the wider patient and societal perspective. This is partially because it does not reflect other dimensions of social value relating to the burden of the disease, the innovation level of interventions and their wider socioeconomic impact [1, 2], therefore acting as an incomplete value metric for cancer treatments and genetic testing [3, 4]. These limitations have led often to the ad hoc and non-systematic use of additional parameters of value by policy-makers which, due to lack of transparency, have given an impression of inconsistency in evidence appraisal and decision-making. Decision controversies however primarily exist because of varying value perspectives, with disagreement being evident among different stakeholders [5]. Therefore, for any decision outcome to be ultimately understood and regarded as “rationally-based”, the application of more comprehensive decision-making procedures of an explicit and transparent nature is required.

Developing alternative methodological approaches for the evaluation of new medicines could therefore potentially overcome such limitations, contributing to a more complete framework for measuring value and making resource allocation decisions. Recently, the use of multiple criteria decision analysis (MCDA) has appeared as a possible methodology to address current limitations of HTA that result from traditional economic evaluation [6–13]. Indeed, one of the conclusions of a recent systematic literature review on MCDA approaches applied in health care, including HTA, was that decision-makers are positive about the potential of MCDA to improve decision-making [14].

However limited studies have produced empirical evidence from real world MCDA applications with the involvement of stakeholders. In this paper we present a case study as proof-of-concept, applying in practice a recently developed MCDA methodological framework [15, 16]. A decision conference workshop was organised with the participation of a wide range of stakeholders for evaluating and ranking a set of drugs for the treatment of metastatic colorectal cancer (mCRC) following first line chemotherapy. We adopted a facilitated decision analysis modelling approach for expert panels [17]. Metastatic colorectal cancer was chosen because of its high severity, the availability of several expensive alternative treatment options, and the fact that it has been the topic of appraisals by several HTA agencies, including to a number by the National Institute for Health and Care Excellence (NICE) in England [18–23].

The methodological details of the case study are extensively provided in the section below. The overall value rankings of the different drugs are presented in the results section, and the limitations of the study together with the challenges encountered are described in the discussion.

## Methods

### Methodological process

An MCDA methodological process was adopted based on Multi-Attribute Value Theory (MAVT) [24, 25] that comprises five distinct phases. These include (a) problem structuring, (b) model building, (c) model assessment, (d) model appraisal, and (e) development of action plans [15]. Further details are provided in the Appendix. A new value framework, the Advance Value Framework, has been proposed as part of which a generic value tree has been developed, incorporating different evaluation criteria for assessing the value of new medicines and introducing a set of MAVT modelling techniques for preference elicitation and aggregation [16]. The new value framework was tested in practice and was operationalised using a decision support software enabling the use of graphics to build a model of values, facilitating both the design phases (a, b) and the evaluation phases of the process (c, d) [26].

### Clinical practice and scope of the exercise (problem structuring)

This is a simulation exercise focusing on identifying and assessing the overall value of second-line biological treatments for mCRC following prior oxaliplatin-based (first line) chemotherapy, by adopting the respective scope from the latest Technology Appraisal (TA) of each technology that has been appraised by NICE (at the time of study design and data collection, February 2015). As part of the technology appraisal process of NICE, clinical and economic evidence from a variety of sources is reviewed to assess the technology’s health benefits (including impact on quality of life and likely effects on mortality), the technology’s costs (focusing on costs to the NHS and personal social services) and the technology’s relation of benefits to costs, or “value-for-money” [27]. Therefore, it should be highlighted that although the remit of NICE is to develop recommendations on the appropriate use of new technologies within the NHS based on their clinical and cost effectiveness, the aim of this exercise is different and relates to assessing a set of treatments for a common disease indication and ranking them based on their value by considering additional types of evidence relating to their benefit. The same or latest available clinical and economic evidence from the corresponding TAs was used to populate the performance of the alternative options across the respective criteria attributes of our value tree, but in addition supplementary evidence was used for value concerns not addressed by NICE. The scope of TA242 was adopted for the cases of bevacizumab, cetuximab and panitumumab [21], whereas the scope of TA307 was adopted for the case of aflibercept [22]. For the case of regorafenib, no sufficient scope details existed in TA334 as the appraisal was terminated early “because no evidence submission was received” from the manufacturer [23], excluding it from the exercise. Further details on the scope of the TAs including the alternative treatments compared and their respective indications are provided in the Appendix.

### Adaptation of the Advance Value Tree for metastatic colorectal cancer (model building)

Overall, we adopted a hybrid approach for the selection of evaluation criteria [15] containing elements both from the “value focused thinking” [28] and “alternative focused thinking” approaches [29].

A generic value tree offering an organised overview of the various value concerns when evaluating new medicines in an HTA context, the Advance Value Tree, has been developed under the auspices of the Advance-HTA project^1^ using a combination of literature reviews and expert consultations [16, 30]. The aim was to identify all the necessary criteria for assessing the value of new medical technologies under a prescriptive decision-aid approach and it was designed in a top-down "value-focused thinking" manner (criteria selected prior identifying the alternative options) [15, 28], generating the building blocks of a comprehensive value function. Ultimately, the resulting value tree is decomposed into five value criteria clusters relating to i) the burden of disease the technology addresses (BoD), ii) the technology’s therapeutic impact (THE), iii) the technology’s safety profile (SAF), iv) the overall innovation level (INN) and, v) the wider socioeconomic impact (SOC):

1 $$ Value=\boldsymbol{f}\left(\boldsymbol{BoD}+\boldsymbol{THE}+\boldsymbol{SAF}+\boldsymbol{INN}+\boldsymbol{SOC}\right) $$

These five clusters of value dimensions were perceived to comprise the critical aspects of value concerns to decision makers for evaluating the value of new medicines as part of HTA from a societal perspective [16], without restricting “value” to the NHS. In addition to scientific value judgments relating to therapeutic impact and safety, the Advance Value Tree allows for the incorporation of social value judgements which might be of interest to key actors and stakeholders of different regions, relating to burden of disease, innovation level and socioeconomic impact, all of which can be captured and measured explicitly.

**Fig. 1:**
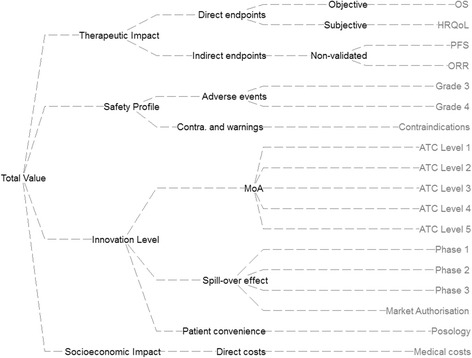
Preliminary value tree for metastatic colorectal cancer (pre-workshop). Abbreviations: Contra. = Contraindications; MoA = Mechanism of action; OS = Overall survival; HRQoL = Health related quality of life; PFS = Progression free survival; ORR = Objective response rate; ATC = Anatomical therapeutic chemical; *Image produced using the Hiview software version 3.2.0.4

The generic value tree was later adapted for the context of mCRC in a bottom-up "alternative-focused thinking" manner (criteria emerged following the comparison of the alternative options) [15, 29]. This adaptation resulted in the preliminary version of the mCRC-specific value tree (Fig. 1). Overall, out of the five criteria clusters of the generic value tree, the burden of disease cluster was removed because it was identical across the alternative treatment options given that all of them were assessed for the same indication (mCRC). The rest criteria clusters were decomposed into nine sub-criteria clusters with a total of 18 criteria attributes. The list of attributes and their respective definitions are shown in Table 1. In arriving at the mCRC-specific attributes and the respective value tree, we strived to adhere to key properties such as preferential independence and non-redundancy in order to ensure their selection is methodologically correct and theoretically robust according to decision theory principles [31].

**Table 1:** Attributes definition and sources of evidence

Cluster	Attribute	Definition	Evidence source		
			Aflibercept + FOLFIRI	Cetuximab	Panitumumab
Therapeutic Impact	Overall survival	The median time from treatment randomisation to death	Van Cutsem et al. 2012 [32]	Price et al. 2014 [33]	Price et al. 2014
	HRQoL	Health related quality of life using EQ-5D score	TA 307 [22]	Hoyle et al. 2013 [35]	Hoyle et al. 2013
	Progression free survival	The median survival time during which patients have not experienced disease progression (using RECIST criteria)	Van Cutsem et al. 2012	Price et al. 2014 [33]	Price et al. 2014
	Objective response rate	The proportion of patients that experience complete response and partial response (using RECIST criteria)	Van Cutsem et al. 2012	Price et al. 2014	Price et al. 2014
Safety Profile	Grade 3 AEs	The proportion of patients experiencing a Grade 3 adverse event	Van Cutsem et al. 2012	Price et al. 2014	Price et al. 2014
	Grade 4 AEs	The proportion of patients experiencing a Grade 4 adverse event	Van Cutsem et al. 2012	Price et al. 2014	Price et al. 2014
	Contra-indications	The existence of any type of contraindication accompanying the treatment	EPAR [39], Prescribing info	EPAR [37], Prescribing info	EPAR [38], Prescribing info
Innovation Level	ATC Level 1	The technology’s relative market entrance in regards to its ATC Level 1 (Anatomical)	WHO ATC index [40]	WHO ATC index	WHO ATC index
	ATC Level 2	The technology’s relative market entrance in regards to its ATC Level 2 (Therapeutic)	WHO ATC index	WHO ATC index	WHO ATC index
	ATC Level 3	The technology’s relative market entrance in regards to its ATC Level 3 (Pharmacological)	WHO ATC index	WHO ATC index	WHO ATC index
	ATC Level 4	The technology’s relative market entrance in regards to its ATC Level 4 (Chemical)	WHO ATC index	WHO ATC index	WHO ATC index
	ATC Level 5	The technology’s relative market entrance in regards to its ATC Level 5 (Molecular)	WHO ATC index	WHO ATC index	WHO ATC index
	Phase 1	The number of new indications for which the technology is investigated in Phase 1 clinical trials	ClinicalTrials.gov [41]	ClinicalTrials.gov	ClinicalTrials.gov
	Phase 2	The number of new indications for which the technology is investigated in Phase 2 clinical trials	ClinicalTrials.gov	ClinicalTrials.gov	ClinicalTrials.gov
	Phase 3	The number of new indications for which the technology is investigated in Phase 3 clinical trials	ClinicalTrials.gov	ClinicalTrials.gov	ClinicalTrials.gov
	Marketing authorisation	The number of new indications for which the technology has gained a marketing authorisation approval	ClinicalTrials.gov	ClinicalTrials.gov	ClinicalTrials.gov
	Posology	The frequency of doses in a given time period in combination with the duration of the administration	EPAR, Prescribing info	EPAR, Prescribing info	EPAR, Prescribing info
Socio-economic Impact	Medical costs impact	The impact of the technology on direct medical costs excluding the purchasing costs of the technology	BNF 69, TA 307, Wade et al. 2013 [34]	BNF 69, TA 242 [21], Hoyle et al. 2013	BNF 69, TA 242, Hoyle et al. 2013

### Evidence considered and alternative treatments compared (model building)

The alternative treatment options compared in the exercise included cetuximab monotherapy (Erbitux ®), panitumumab monotherapy (Vectibix ®), and aflibercept (Zaltrap ®) in combination with FOLFIRI chemotherapy. Although there is published evidence for the efficacy of cetuximab in combination with chemotherapy, bevacizumab in combination with non-oxaliplatin-based chemotherapy, and regorafenib monotherapy as treatment options, we did not include these treatments in the exercise because there was absence of relevant clinical evidence submitted to NICE as part of their respective TAs [21, 23].

Overall, evidence sources used to populate the preliminary model included two randomised clinical trials (RCTs) [32, 33], the respective NICE TAs [21, 22], the NICE Evidence Review Group (ERG) reports [34] or any related peer review publications [35, 36], summaries of product characteristics (SPCs) available through EMA’s European Public Assessment Reports [37–39] (or highlights of prescribing information leaflets), Anatomical Therapeutic Chemical (ATC) classification system indexes through the portal of the WHO Collaborating Centre for Drug Statistics Methodology [40], and ClinicalTrials.gov listings [41]. The sources of evidence used for identifying the performance of the treatment options across the criteria attributes are shown in Table 1. It should be noted that among the two RCTs used for populating the performance of the treatments across the clinical attributes, one was a head to head trial directly comparing cetuximab versus panitumumab (*ASPECCT* trial) [33] and the other one comparing aflibercept in combination with FOLFIRI versus placebo with FOLFIRI (*VELOUR* trial) [32]. More details on the clinical evidence considered are provided in the Appendix.

### Setting attribute ranges and reference levels (model building)

As part of model building, we selected attribute ranges that were encompassed within minimum (min) and maximum (max) levels. Within the min-max attribute range we defined “lower” (x_l) and “higher” (x_h) reference levels to act as benchmarks for the preference value scores of 0 and 100 respectively, needed for the construction of criteria value functions and elicitation of relative weights (these are interval scales and thus the importance of setting up clear bounds for each attribute). Incorporation of such intermediate reference levels rather than extreme reference levels at the limits of the value scale can protect against inaccuracies emerging from potential non-linearity in value at scale’s limits [42] and could ensure that the value scale has enough granularity to distinguish the treatments. As a result, value scores could possibly be negative or higher than 100 with v(x_lower_) = 0 and v(x_higher_) = 100, essentially conducting a linear transformation which is admissible to an interval scale such as a value scale. The methodological basis for setting the attribute ranges and reference levels is described in the Appendix. A list of all attributes’ “lower” and “higher” reference levels together with their basis of selection, as shaped before the workshop is provided in Table 2.

**Table 2:** Pre-workshop attribute reference levels and basis of selection

Cluster	Attribute	Metric	Lower level	Basis	Higher level	Basis
Therapeutic Impact	Overall survival	months	0	Minimum limit of the scale	6.2	BSC
	HRQoL	utility score (EQ-5D)	0.6	Lower score used for progressive state in TA307 [22]	0.75	BSC
	Progression free survival	months	0	Minimum limit of the scale	1.9	BSC
	Objective response rate	% of patients	0	Minimum limit of the scale	11	FOLFIRI + Placebo (VELOUR trial) [32]
Safety Profile	Grade 3 AEs	% of patients	68	10% higher than the worst performing option	32	Median of BSC (AMGEN trial) [65] and FOLFIRI + Placebo (VELOUR trial)
	Grade 4 AEs	% of patients	24	10% higher than the worst performing option	10	Median of BSC (AMGEN trial) and FOLFIRI + Placebo (VELOUR trial)
	Contra-indications	types of contra- indications	Lower expected benefit and higher expected risk	Minimum limit of the scale	Lower expected benefit	Median of options
Innovation Level	ATC Level 1	relative market entrance	5th	Minimum limit of the scale	4th	Median of options
	ATC Level 2	relative market entrance	5th	Minimum limit of the scale	4th	Median of options
	ATC Level 3	relative market entrance	5th	Minimum limit of the scale	3rd	Median of options
	ATC Level 4	relative market entrance	5th	Minimum limit of the scale	1st	Median of options
	ATC Level 5	relative market entrance	5th	Minimum limit of the scale	1st	Median of options
	Phase 1	number of new indications	0	Minimum limit of the scale	17	Median of options
	Phase 2	number of new indications	0	Minimum limit of the scale	55	Median of options
	Phase 3	number of new indications	0	Minimum limit of the scale	18	Median of options
	Marketing authorisation	number of new indications	0	Minimum limit of the scale	2	Median of options
	Posology	duration of administration & frequency of doses	Many hours, every 2 weeks	Minimum limit of the scale (worst performing option)	Up to an hour, every 2 weeks	Maximum limit of the scale (best performing option)
Socio-economic Impact	Medical costs impact	GBP (£)	7,086	10% higher than the worst performing option	4,589	Median of options

### Decision conference (model assessment and appraisal)

The model assessment and model appraisal phases of the exercise took place through a facilitated workshop with key stakeholders and experts, taking the form of a decision conference [43], organised by the authors and hosted at the London School of Economics and Political Science (LSE) on 30th of April 2015. Decision conference could be defined as “a gathering of key players who wish to resolve important issues facing their organisation, assisted by an impartial facilitator who is expert in decision analysis (DA), using a model of relevant data and judgements created on-the-spot to assist the group in thinking more clearly about the issues” [44] (p.54); see also [17]. Typical stages of decision conference workshops include exploring the issues, structuring and building the model, exploring the model and agreeing on the way forward. In our study, the first two stages were to a great extent informed by preparatory work that had been conducted before the workshop, involving extensive literature reviews.

Background material introducing the scope of the exercise in more detail was sent to the participants one week before the workshop. On the day of the workshop, the model was presented to the participants and was revised cluster by cluster in real time through a facilitated open discussion. It should be highlighted that the aim of the model in such MCDA evaluation contexts is to act as an aid for the group to interact and think about the decision problem constructively, rather than to provide the “correct” answer [44, 45]. An iterative and interactive model-building process was adopted, where debate was encouraged and differences of opinion actively sought. Generally, overall agreement was reached in regards to criteria inclusion and exclusion; in the few instances where this was unattainable, criteria were left in the model for their impact to be tested as part of the sensitivity analysis stage, where distinctive viewpoints were finally resolved.

The composition of the group’s expertise and the numbers of the different stakeholders were decided based on the structure of the past NICE committees responsible for the appraisals of the alternative treatments [18, 20–22]. We aimed to involve a small group between seven and 15 participants; these group sizes have been shown to be sufficient because they tend to preserve individuality while also allowing efficient group processes to emerge, as they are small enough to be able to work towards agreement, but large enough to represent all major perspectives [46]. In total, 13 participants were involved, their areas of expertise and type of affiliation are shown in Table 3. More details about the decision conference can be found in the Appendix.

**Table 3:** List of decision conference participants

Participant	Expertise	Affiliation
1	Medical oncologist - CRC expert	NHS Trust/Teaching hospital
2	Medical oncologist - CRC expert	NHS Trust
3	Consultant - community paediatrician	NHS Trust/HTA agency
4	Public health expert	Academia
5	Pharmacist	Independent
6	Health economist	Academia
7	HTA expert	Academia
8	Health economist	Academia
9	HTA expert	Academia
10	Medical statistician	Academia
11	Patient	Independent
12	Patient carer	Independent
13	Patient advocate	Charity

### MCDA technique (model assessment and appraisal)

The selection of scoring, weighting and aggregating techniques depends on the characteristics of the particular decision-making problem under consideration, as for example the level of analytical precision required for the evaluation of the alternative interventions and the cognitive burden expected to be posed to key actors and decision-makers [13]. The pros and cons of different MCDA modelling techniques in health care evaluation and more precisely on the decision-making process and outcomes generally remain unknown, therefore their selection represents an important area demanding further research.

Value preferences can be elicited using different questioning protocols. A value measurement method based on pairwise qualitative comparisons is MACBETH (Measuring Attractiveness by a Categorical Based Evaluation Technique), an approach using qualitative judgments about the difference of attractiveness between different pairs of attribute levels [47, 48]. In this study we decided to test MACBETH because of its strong theoretical foundations [49], numerous applications for real world problems [50, 51] and expected usefulness in HTA settings. As part of MACBETH, semantic judgments made either by individuals or groups are converted into a cardinal scale, providing a simple, constructive and interactive approach with good prospects for facilitating the preference elicitation process of groups, such as in technology appraisal committee settings.

We adopted the typical simple additive (i.e. linear average) aggregation approach, where the overall value V(.) of an option *a* is given by the following Equation [52]:

2 $$ V(a)=\sum_{i=1}^m{w}_i\ {v}_i(a) $$

Where *m* is the number of criteria (attributes), and *w*_*i*_ *v*_*i*_ *(a)* the weighted partial value function of criterion *i* for option *a*. This function V(.)_ is denominated a multi-attribute value function [25].

We operationalised the methodology using M-MACBETH [53], a decision support system based on the MACBETH approach, to elicit the value preferences of workshop participants and more precisely to build the value tree, elicit value functions for the different attributes, assign attribute relative weights through a qualitative swing weighting approach, aggregate the preference value scores and weights using an additive aggregation (i.e. simple additive model) to derive overall weighted preference value (WPV) scores, and conduct sensitivity analysis [54]. Besides a consistency check between the qualitative judgments expressed that is automatically provided by the software, a second consistency check was performed manually by the facilitator to ensure that an interval scale is obtained, i.e. validate the cardinality of the scale. This took place by comparing the sizes of the intervals between the suggested scores and inviting participants to adjust them if necessary [55], an essential requirement for aggregation using simple additive value models. More technical details on MACBETH are provided in the Appendix.

### Costs calculation

Drug costs were calculated according to prices (excl. VAT), pack sizes and dosage strengths as found in the British National Formulary (BNF 69), and the recommended dosage and treatment duration as reported in the respective NICE technology appraisals [21, 22]. Vial wastage was assumed in all calculations. Drug administration costs for cetuximab and panitumumab were kept consistent with Hoyle et al. [36] and administration costs for aflibercept plus FOLFIRI consistent with the respective ERG Report [34].

## Results

### Criteria validation and amended value tree for metastatic colorectal cancer

**Fig. 2:**
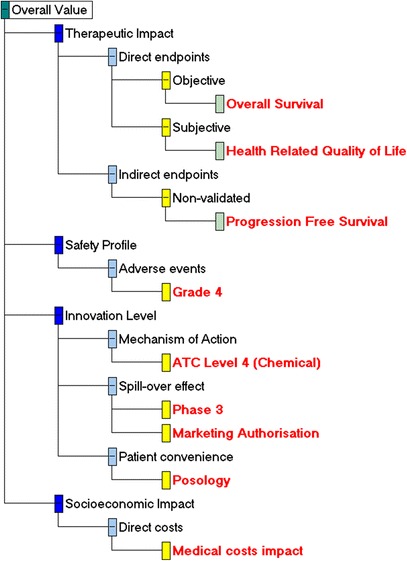
Final Value Tree for metastatic colorectal cancer (post-workshop). * Image produced using the M-MACBETH (beta) software version 3.0.0

The final version of the value tree, as emerged following the open discussion with the participants in the workshop is shown in Fig. 2. In total, nine out of the 18 attributes were removed from the value tree because they were judged from the participants to be non-fundamental for the scope of the exercise, resulting in a value tree of half its original size. Importantly, no criteria were deemed to be missing. In the therapeutic impact cluster, the Objective Response Rate (ORR) attribute was removed. In the safety profile cluster, the contra-indications attribute was removed and the Grade 3 Adverse Events (AEs) and Grade 4 AEs attributes were proposed to be aggregated into a single attribute; however this aggregation required a significant modelling iteration, and due to time constraints it was decided to exclude the Grade 3 AEs attribute and only include Grade 4 AEs for the purpose of the simulation exercise. In terms of the innovation cluster, participants had mixed views. Consensus was reached for the ATC Level (L)5, Phase 1 and Phase 2 attributes to be removed, and for the ATC L4 to be included as a binary variable (i.e. first entrance in the chemical class vs. second or subsequent entrance in the chemical class); however strong disagreement existed on whether to include Phase 3 and Marketing Authorisation attributes, with half the participants in favour of and half the participants against their inclusion. As a result, both of the attributes were left in the model and their impact was then tested at the end of the workshop as part of the sensitivity analysis stage.

### Validation of attribute ranges and reference levels

Another important amendment in the model included a change in the definitions of the “lower” and “higher” reference levels, which define the 0 and 100 scores in the value functions and act as anchors for swing weighting. For the case of clinical attributes (i.e. therapeutic impact and safety profile attributes), a consensus was reached by the group that the “lower reference” level should actually correspond to the “satisfactory performance” (proxied by Best Supportive Care, BSC) rather than the “worst performance” plausible. As a result, the “lower reference” level was switched to the previously defined “higher reference” level (i.e. satisfactory performance), and the “higher reference” level was set equal to the maximum level. The newly-defined attribute levels were therefore: i) the “lower reference” level (i.e. BSC-based satisfactory performance); the “higher reference” level (equal to the maximum, best performance plausible, level), and the minimum level (i.e. worst performance plausible). In doing so, the amended “lower” and “higher” reference levels were now corresponding to “satisfactory performance” (proxied by BSC) and “best performance” respectively, with options performing worse than the “satisfactory” level getting a negative score, and all options obtaining less than 100 score.

As a consequence, a similar change was introduced for the case of - now single - safety profile attribute (Grade 4 AEs), with the “lower reference” level being defined based on “satisfactory performance”, the “higher reference” level based on “best performance” (i.e. minimum limit of the scale) and the minimum level remaining the same.

For the case of innovation attributes, the “higher reference” levels were set equal to the “best performance” levels, with the “lower reference” levels remaining the same (equal to the worst performance). Similarly, for the case of the socioeconomic impact attribute (impact on direct medical costs) the “higher reference” level was also set equal to the “best performance” level, with the “lower reference” level remaining the same (equal to the worst performance).

**Fig. 3:**
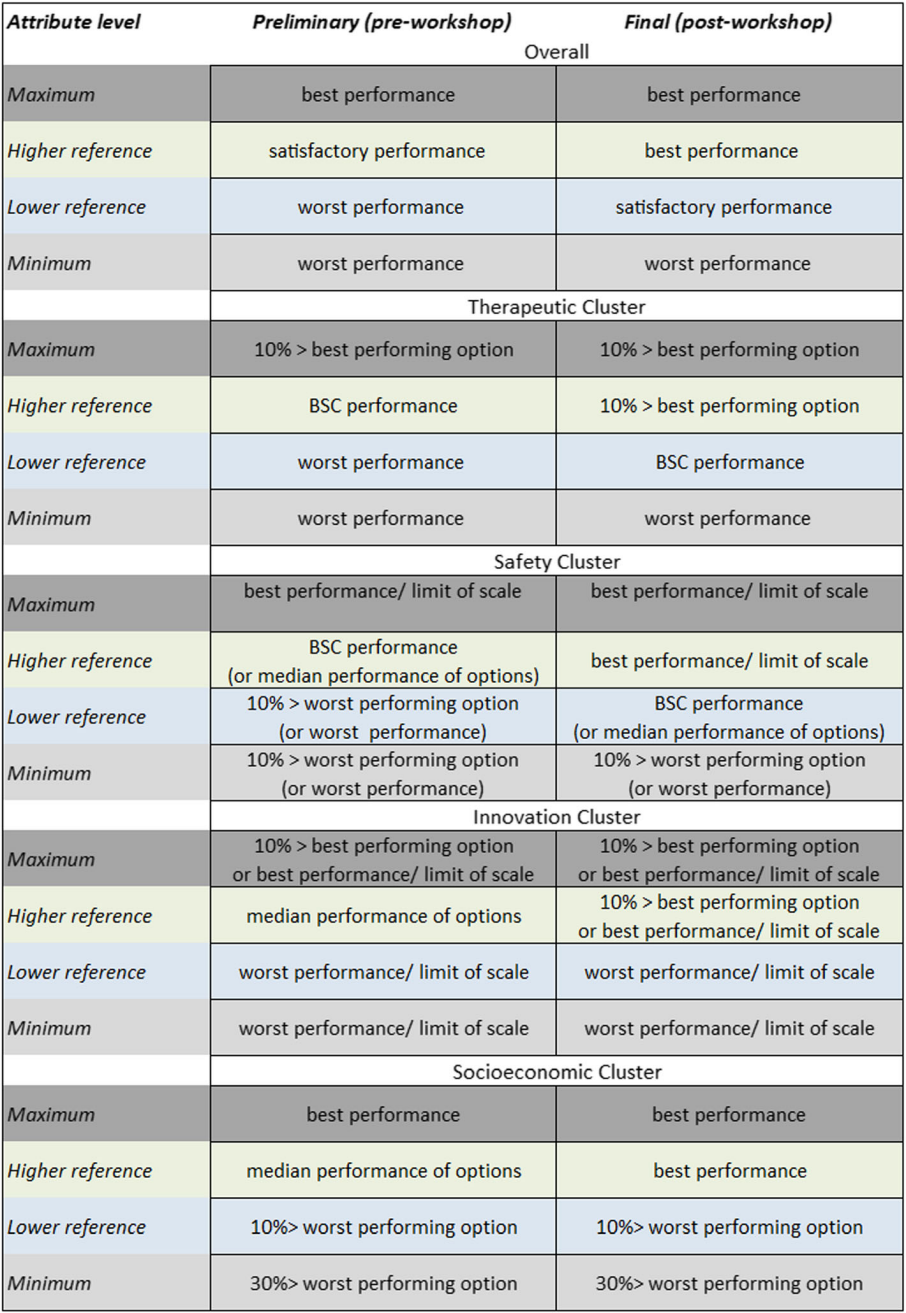
Changes in the definitions of the attribute reference levels, pre- and post- workshop

The arising changes in the attribute reference level definitions, before and after the workshop for each of the criteria clusters are shown in Fig. 3, and the final list of attributes’ “lower” and “higher” reference levels, together with their basis of selection are provided in Table 4.

**Table 4:** Post-workshop attribute reference levels and basis of selection

Cluster	Attribute	Metric	Lower level	Basis	Higher level	Basis
Therapeutic Impact	Overall survival	months	6.2	BSC	14.9	10% higher than the best performing option
	HRQoL	utility score (EQ-5D)	0.75	BSC	0.9	10% higher than the best performing option/ general population
	Progression free survival	months	1.9	BSC	7.6	10% higher than the best performing option
Safety Profile	Grade 4 AEs	% of patients	10	Median of BSC arm from AMGEN trial and placebo + FOLFIRI arm from VELOUR trial	0	Maximum limit of the scale
Innovation Level	ATC Level 4	relative market entrance	≥2nd	Minimum limit of the scale, binary variable	1st	Maximum limit of the scale, binary variable
	Phase 3	number of new indications	0	Minimum limit of the scale	21	10% higher than the best performing option
	Marketing authorisation	number of new indications	0	Minimum limit of the scale	3	10% higher than the best performing option
	Posology	duration of administration & frequency of doses	Many hours, every two weeks	Minimum limit of the scale (worst performing option)	Up to an hour, every two weeks	Maximum limit of the scale (best performing option)
Socio-economic Impact	Medical costs impact	GBP (£)	7,086	10% higher than the worst performing option	0	BSC

### Options performance, criteria weights and overall preference value rankings

Two examples of value judgements matrices and their conversion into a linear and non-linear value function for the case of the Overall Survival (OS) and Health Related Quality of Life (HRQoL, measured by the EQ-5D score) attributes respectively are shown in the Appendix.

**Fig. 4:**
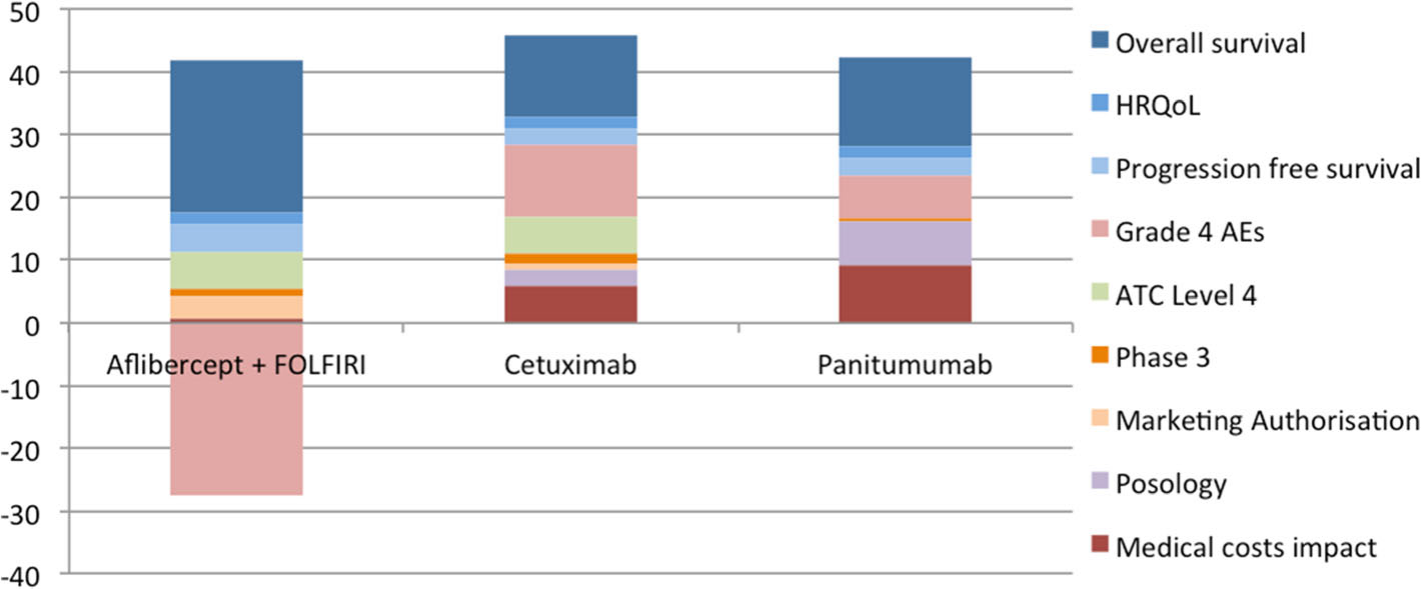
Stacked bar plot of treatments’ individual weighted preference value (WPV) scores across all attributes. Abbreviations: HRQoL = Health related quality of life; AE = Adverse Event; ATC L4 = Anatomical therapeutic chemical level 4

The performance of the options across the different attributes together with the “lower” and “higher” reference levels are shown in Table 5. The different columns correspond to the performance of the different options (including the two reference levels), across the respective attributes shown in the rows. The overall WPV scores for all options across the different attributes, together with the respective attribute baseline weights are shown in Table 6; similarly to Table 5, the different columns correspond to the preference value scores of the different options (including the two reference levels), across the respective attributes shown in the rows. Cetuximab scored the highest overall WPV score of 45.7, followed by panitumumab with an overall WPV score of 42.3. Aflibercept in combination with FOLFIRI was ranked last with an overall WPV score of 14.4, partially due to its performance on Grade 4 AEs (21%) which lied below the lower reference level of the value scale (10%), producing an absolute preference value score of −117.9 and a weighted preference value score of −27.4. A stacked bar plot of the WPV scores of the alternative treatments across the attributes is shown in Fig. 4.

**Table 5:** Options performance across the criteria attributes

Attribute	Metric	Lower level	Aflibercept + FOLFIRI	Cetuximab	Panitumumab	Higher level
Overall survival	months	6.2	13.5	10	10.4	14.9
HRQoL	utility (EQ-5D)	0.75	0.78	0.78	0.78	0.9
Progression free survival	months	1.9	6.9	4.1	4.4	7.6
Grade 4 AEs	% of patients	10	21	5	7	0
ATC L4	relative market entrance	2nd	1st	1st	2nd	1st
Phase 3	# of new indications	0	18	19	7	21
Marketing Authorisation	# of new indications	0	3	1	0	3
Posology	duration & frequency	hours, every 2 weeks	hours, every 2 weeks	1 hour, every week	≤1 hour, every 2 weeks	≤1 hour, every 2 weeks
Medical costs impact	GBP (£)	7,086	6,738	4,589	1,940	0

**Table 6:** Overall weighted preference value (WPV) scores, individual preference value scores, relative weights, costs and cost per unit of value

	Lower level	Aflibercept + FOLFIRI	Cetuximab	Panitumumab	Higher Level	Relative Weights
**Overall WPV score**	**0.0**	**14.4**	**45.7**	**42.3**	**100.0**	**100**
Overall survival	0.0	83.9	44.4	48.9	100.0	29
HRQoL	0.0	15.0	15.0	15.0	100.0	13
Progression free survival	0.0	90.3	51.4	55.6	100.0	5
Grade 4 AEs	0.0	−117.9	50.0	30.0	100.0	23
ATC L4	0.0	100.0	100.0	0.0	100.0	6
Phase 3	0.0	50.0	66.7	19.4	100.0	2
Marketing Authorisation	0.0	100.0	30.0	0.0	100.0	3
Posology	0.0	0.0	37.5	100.0	100.0	7
Medical costs impact	0.0	7.0	50.0	78.9	100.0	12
*Costs (£)*		29,400	18,000	27,000		
*Cost per unit of value*		2,046	394	638

**Fig. 5:**
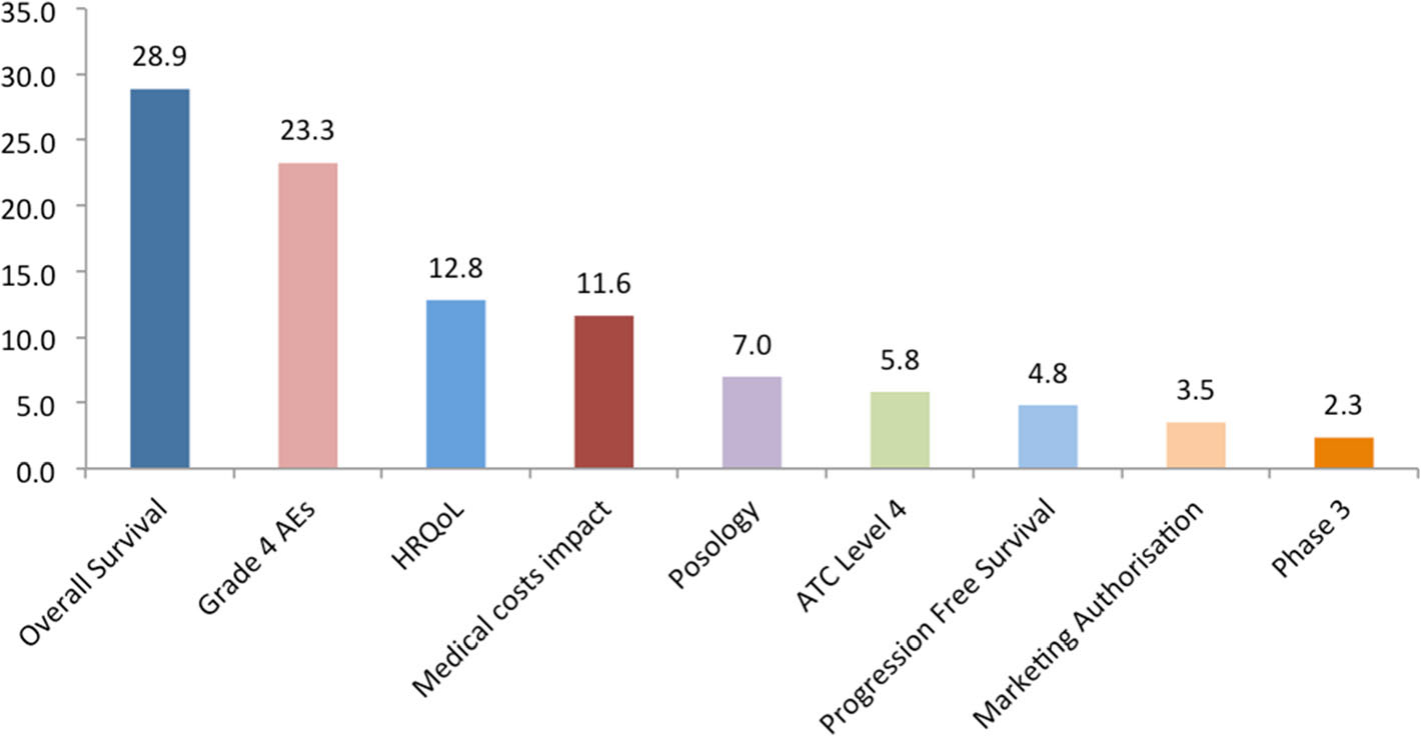
Criteria weights histogram. Abbreviations: AE = Adverse Event; HRQoL = Health related quality of life; ATC L4 = Anatomical therapeutic chemical level 4

The relative weights assigned to the different attributes are shown in Fig. 5. The criteria are ranked based on their relative magnitude, ranging from relatively more important criteria to relatively less important criteria (from left to right across the x-axis), taking into account the “lower” – “higher” ranges of the attributes. The OS and Grade 4 AEs attributes were assigned a relative weight totaling more than the relative weights of all other attributes together, i.e. 52%. Out of 100, the therapeutic impact cluster (three attributes) totaled overall a relative weight of 47, the safety profile cluster (single attribute only) a relative weight of 23, the innovation level cluster (four attributes) a relative weight of 19, and the socioeconomic impact cluster (single attribute only) a relative weight of 12.

### Value for money analysis

**Fig. 6:**
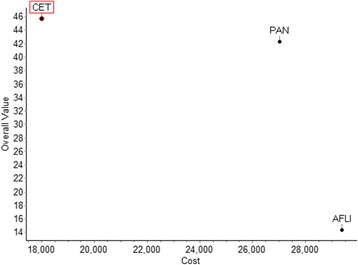
Cost benefit plot of overall weighted preference value (WPV) scores versus costs. Abbreviations: CET = cetuximab; PAN = Panitumumab; AFLI = Aflibercept (plus FOLFIRI). * Image produced using the M-MACBETH (beta) software version 3.0.0

By incorporating the total purchasing costs of the different drugs (including their administration costs), their overall WPV scores versus their costs can be plotted, taking the form of an MCDA cost-benefit plane (Fig. 6). Using purchasing costs of treatments separately to their overall value scores is based on the rationale that only attributes of benefit should act as criteria in multi-criteria models [56]. However, a treatment’s impact (i.e. net effect) on resources, ranging from medical related to productivity gain, is usually an important value concern for decision-makers and could therefore act as a relevant objective or criterion in the analysis. For this reason, “impact on costs” in relation to a relevant benchmark comparator rather than absolute costs could be considered as part of the valuation, being exclusive of the purchasing costs of the drugs themselves which could then be used as the cost component to calculate an MCDA cost-benefit metric in tandem to their value scores [16]. By using rounded up total cost figures of £18,000 for cetuximab (£12,824 drug cost and £5,191 administration cost), £27,000 for panitumumab (£23,643 drug cost and £3,374 administration cost), and £29,400 for aflibercept in combination with FOLFIRI (£17,750 drug cost and £11,630 administration cost), and dividing them with overall WPV scores, their costs per MCDA value unit were calculated to be £394, £638, and £2,046 respectively (Table 6). Assuming the use of a common treatment comparator, the incremental cost value ratio (ICVR) of the different health care interventions could be estimated, a form of an MCDA cost-benefit ratio. Therefore, in terms of value-for-money, aflibercept in combination with FOLFIRI is shown to be dominated by panitumumab, both of which are shown to be dominated by cetuximab which is associated with the highest overall WPV score and the lowest cost.

NICE’s conclusion about the three technologies was that none of them should be recommended for use within NHS because they either do not represent a cost-effective use of NHS resources (for the case of cetuximab and panitumumab monotherapies) [21], or because of significant uncertainty around the extrapolation of overall survival and a higher than normally acceptable maximum incremental cost effectiveness ratio (ICER) range (for the case of aflibercept in combination with chemotherapy) [22]. Interestingly, if the efficiency rankings of the three treatments based on their ICERs (i.e. incremental cost per QALY) were to be compared versus their respective rankings based on their cost per MCDA value unit, a difference would be observed as aflibercept in combination with FOLFIRI produced the lowest ICER (£51,000) [22], followed by cetuximab (around £90,000) and then by panitumumab (>£110,000) [21]. This discrepancy in efficiency rankings according to which aflibercept in combination with FOLFIRI came up third rather than first could partially be explained due to the relatively low value score of the treatment in regards to Grade 4 AEs (−118) in combination with its relatively large weight (0.23) which could however be perceived as a study limitation (see the discussion under the Limitations and challenges section).

### Sensitivity and robustness analysis

Deterministic sensitivity analysis was conducted to address parameter uncertainty by exploring the impact of baseline weight changes on the ranking of the options (figures shown in the Appendix). In order for panitumumab to become better ranked than cetuximab any of the following changes in baseline weights would be needed: OS from 28.9 to 59.8, PFS from 4.8 to 47.7, Grade 4 AEs from 23.3 to 7.5, ATC L4 from 5.8 to 2.5, Posology from 7 to 11.8, or Medical costs impact from 11.6 to 21.0. Similarly, for aflibercept plus FOLFIRI to become better ranked than cetuximab any of the following changes in baseline weights would be needed: OS from 28.9 to 60.3, PFS from 4.8 to 47.3, Grade 4 AEs from 23.3 to 5.6, or Marketing Authorisation from 3.5 to 33.3. Finally, for aflibercept plus FOLFIRI to become better ranked than panitumumab any of the following changes in baseline weights would be needed: OS from 28.9 to 60.4, PFS from 4.8 to 47.2, Grade 4 AEs from 23.3 to 5.4, ATC L4 from 5.8 to 26.4, Phase 3 from 2.3 to 49.0, or Marketing Authorisation from 3.5 to 24.6.

Therefore, conclusions were fairly robust as treatment rankings were not influenced by changes of 50% or less on any of the baseline normalised weights, the most sensitive attributes being Posology and Medical costs impact attributes on the cetuximab versus panitumumab comparison (requiring a 69% and 81% change respectively for panitumumab to become better ranked), with changes of at least up to 100% on the remaining baseline normalised weights exerting no impact on the results.

The robustness of the results was also tested by conducting 8-way sensitivity analysis in the reference levels of the attributes using the respective function of the M-MACBETH software (“Robustness analysis”), which showed that a simultaneous change of up to 5 value points across all of the attribute reference levels would not impact the ranking of the alternative treatments (figure in the Appendix).

However other types of uncertainty might exist, such as stochastic uncertainty, structural uncertainty and heterogeneity, which could be addressed through more advanced statistical approaches, including probabilistic sensitivity analyses, Bayesian frameworks, fuzzy set theory or grey theory [57]. For example, uncertainty associated with the performance of the options due to sampling variation of clinical studies, or with the criteria weights due to inability to derive or agree on weights, might make the application of point estimates inappropriate in which case stochastic multi-criteria acceptability analysis (SMAA) might be preferred [58].

## Discussion

This simulation exercise adopts an MCDA methodological process for the context of HTA [15] and tests a newly developed value framework (Advance Value Framework) in practice through a decision conference approach [52]. The methodological process we adopted is generally in alignment with recent good practice guidelines on the use of MCDA for health care decision-making, in respect to design, implementation and review of the analysis [13].

Overall, a set of different treatment options for the indication of mCRC at second-line were assessed and ranked based on their overall WPV scores. These scores acted as value metrics or value indexes, comprised of the performance of the alternative treatment options against an explicit set of criteria while adjusting for the relative importance of these criteria, as reflected by the preferences of the group. Finally, incorporation of drug costs (purchasing and administration costs) enabled the production of “cost per unit of value” ratio estimates while revealing the dominance of one treatment. Assuming that the participants of the workshop formed a committee group responsible for choosing or recommending the funding of one of the three alternative options as part of a one-off decision, this treatment could be a rational choice for the group assuming that the respective budget needed is available. It should be clear that the decision context considered and problem scope addressed by this study is not in line with the current NICE remit, which is that of repeated decisions, as part of which an intervention is evaluated in terms of whether it provides good value for money given the opportunity cost to invest in other interventions across different disease areas. Therefore, while in this exercise the top-ranked option would be recommended, in the case of NICE it might be the case that none of the options is recommended unless they represent an efficient allocation of resources across the total NHS budget.

### Strengths and opportunities

Among the biggest benefits of the methodology adopted as reflected through this particular experimental application are the explicit incorporation of multiple benefit dimensions, some of which are possibly hard-to-measure but proved important nevertheless and the elicitation of value trade-offs between them. A more detailed discussion around the advantages of the value framework from a decision-making perspective is described in the section below (Practice and policy implications).

A central strength of the methodology as experienced through this case study is the development of the evaluation model with a group of relevant stakeholders (health care professionals, methodology experts, patients), which proved to be essential for creating a shared understanding of what constitutes value in this decision context. This was evident across all its phases, ranging from model-building to model appraisal, playing a profound role across all the stages.

Starting with criteria selection, by sharing participants’ views and opinions among the group while seeking a consensus approach, the original version of the value tree and its criteria were validated, amending its contents and leading to the exclusion of some attributes that seemed non-fundamental or irrelevant. For example *Objective Response Rate (ORR)*, the sum (i.e. proportion) of patients that experience complete response and partial response (using the RECIST criteria), which was originally included in the value tree was decided to be removed because of irrelevance. Initially, the clinical view was raised that stable cancer (i.e. non-responding) might be just as good of an outcome as tumour shrinking. Although the argument was expressed that in theory *ORR* could help into controlling symptoms better, there is no firm evidence for this in the literature. The *HRQoL* and *Grade 4 AEs* measures are instruments designed for the assessment of symptoms and *ORR* gives no additional value. Thus, its inclusion in this regard would even entail double counting effects. In turn, it was suggested that *ORR* is primarily designed for measuring patient response and treatment efficacy under the settings of new drug development and not a major guide to clinical practice in the setting of advanced disease. With regard to clinical practice, the use of *PFS* as a metric could be perceived more complete and reflective.

During model assessment and the elicitation of preferences through value functions, the structured discussion as facilitated by one of the authors enabled the representation of all the different perspectives for the purpose of valuation. Although occasionally some of the participants might at first have had opposing views and beliefs in regards to their preference judgments, in most of the cases these conflicts were terminated or defused following extensive discussions. An example would be the elicitation of the *Overall Survival* (*OS)* value function which started with contrasting perspectives on how to assess additional months of life, but following far-reaching dialogues around the added value of different life increments, an agreement was established that each additional month of life was associated with an equal magnitude of value, as revealed through a linear value function.

The systematic assessment of all types of evidence together enabled the identification of strengths and weaknesses for each treatment, which in turn could be used to influence their use under clinical practice, or even support their design and improvement as part of the clinical development process. For example, although aflibercept in combination with FOLFIRI (afli + FOL) was associated with the highest score in *OS*, overall it ranked last, partially because of a highly negative score in *Grade 4 AEs* (for more information see below the Limitations and challenges section). Assuming that such an analysis and discussion were conducted at an earlier stage of the product’s life cycle, for example during Phase 2 clinical trials, these insights could possibly influence future aspects of clinical development by inducing changes into lead design and drug formulation with the view to enhance the envisaged performance characteristics perceived to be outranked or disadvantaged.

Another benefit of the evaluation we conducted was a clear separation between the performance of the treatments and their valuation, based respectively on the availability of evidence across the attributes and the establishment of value increments within criteria and value trade-offs across criteria, with the latter being amenable to sensitivity analysis. The explicit modelling of preferences and values represented how much the group valued incremental performances in each attribute, as well as their priorities for the different criteria, represented by the weights. This separation allowed us to assess the robustness of results for preferences variations. For example, sensitivity analysis at the end of the workshop in respect to the baseline weights of the innovation attributes, for which some of the participants did not fully agree with their elicited relative importance, assured the authors and the participants that the ranking of the treatments was not sensitive to minor variations along their range (last column of Table 6).

### Limitations and challenges

Results should be interpreted with caution. It should be clear that this is a simulation exercise illustrating the application of a new value framework in practice with the results produced not intended to inform policy making in this instance. For this to occur, the clinical evidence used to inform the performance of the treatments across the Therapeutic Impact and Safety Profile clusters should ideally come from head to head clinical trials directly comparing all treatment of interest. Alternatively, relative treatment effects should be estimated through indirect treatment comparisons making use of indirect evidence through a common comparator, or network meta-analysis such as mixed treatment comparisons making use of all available evidence, both direct and indirect [59]. Instead, a limitation in this study is that we used un-synthesised clinical evidence coming from different clinical trials which did not have a common comparator: a head to head clinical trial directly comparing two of the three treatments (*ASPECCT* trial for cetuximab vs panitumumab) and another clinical trial comparing the third treatment with placebo (*VELOUR* trial investigating aflibercept with FOLFIRI vs placebo with FOLFIRI). In real world evaluations aiming to inform policy-making, an evidence synthesis step should be conducted together with evidence collection as part of the model building phase. An example would be the application of an SMAA approach for assessing the comparative benefit-risk of different statins for use in primary prevention [60], which used comparative effects based on evidence that had been originally collected as part of three meta-analyses [61–63], or the combination of SMAA with a network meta-analysis for assessing the comparative benefit-risk of second-generation antidepressants and placebo [64].

Among one of the main challenges was setting the “higher” and “lower” reference levels on each attribute, based on which treatment scores were derived. For example, as described above, in the case of Grade 4 AEs, a “higher” reference level was set equal to the minimum natural limit of the scale (i.e. 0%) that was regarded as an “ideal” level. However, this level could be perceived as extremely optimistic, or “too good to be true”. Possibly more important though in terms of Grade 4 AEs attribute’s impact on the scoring of the alternative treatments was the definition of the “lower” reference level as this influenced the negative partial value score observed for one of the treatments and consequently its overall WPV score. The lower reference level of 10% adopted was regarded as “satisfactory” performance and was derived using the median of the BSC comparator arm of panitumumab’s pivotal clinical trial (*AMGEN* trial) [65], and the placebo plus FOLFIRI comparator arm of aflibercept’s pivotal clinical trial (*VELOUR*) [32]. As a result, aflibercept in combination with FOLFIRI produced a negative partial value score in Grade 4 AEs because its performance was less preferred than the lower reference level. Although we tried to be as objective as possible when setting the reference levels, others might have ended up with different anchor points. However such expected differences most probably would have been of a minor impact, without necessarily affecting the overall valuation of the treatments. The final reference levels adopted were decided following the feedback we received during the workshop. Liaising with a range of experts during the model-building phase for ensuring the choice of relevant reference levels, before the elicitation of preferences and the model assessment phase, seems to be a necessary step for ensuring good practice and robust results.

Another challenge related to the use of the EQ-5D instrument to measure HRQoL (given the lack of other relevant evidence), which is associated with two issues. Firstly, by definition it is an aggregate measure so it does not allow to make value trade-offs among its different dimensions, and, secondly, it already captures the preferences of the general public so the use of unweighted health states might be more appropriate. Therefore, it should be acknowledged that MCDA does not act as a panacea for challenges relating to appropriate evidence collection on patient experience but mainly as a tool to understand, construct and analyse their preferences on already existing evidence.

Another issue would be the evaluation of clusters where participants have less experience or knowledge. For example, during the evaluation of the *HRQoL* attribute, some of the participants had difficulties in comprehending the differences in value between the different EQ-5D index scores. However, feedback from clinicians and patients helped the rest of the group to understand the relative differences across health states so that they could express their preferences. Although such an input proved crucial and, to a large extent, satisfactory to the information prerequisites of the group, it would be advantageous if the EQ-5D index scores were accompanied with qualitative descriptions to illustrate the impact to patients’ quality of life.

An additional limitation would relate to the technical difficulties associated with ensuring that all attributes possess the required theoretical properties for a multi-criteria evaluation, as for example that they are preference independent if used in additive models such as the current one; alternatively, attributes might have to be combined together or multiplicative models might have to be used instead. Furthermore, although in the present case study only the *HRQoL* of the stable disease state is assessed, mainly because none of the treatments is assumed to have any effects during the progressive disease state [22, 36], in other diseases this might not hold true.

### Practice and policy implications

Implementation of MCDA methodologies and their linkage to policy-making could take place in the form a supplementary “incremental” mode to cost-effectiveness analysis (CEA) adjusting the ICER through the incorporation of additional benefit dimensions, or in the form of a pure “clean-slate” mode where value is derived de novo (i.e. from the beginning) without the use of CEA [7, 15].

Both approaches are associated with different pros and cons and as a result the choice between the two should be made depending on the decision context of interest, while taking into account the current evaluation guidelines in place and the flexibility of the decision-makers. For example, the application of an MCDA approach in alignment with a supplementary or “incremental” mode might enable an easier exploration and implementation by decision-makers in real-world settings. Assuming the proposed methodological process is adhered to, as part of a pure or “clean-slate” approach for use in HTA, the application of the Advance Value Framework presents a number of potential advantages to decision-makers in the context of HTA and the wider context of Value Based Assessment compared with currently used HTA approaches such as economic evaluation techniques.

Firstly, it acts as an instrument of more complete value assessment leading to improved comprehensiveness given the explicit incorporation of multiple criteria and construction of value judgements on the performance of alternative options that can help decision-makers to construct their overall value concerns. Secondly, assignment of quantitative criteria weights can reflect differences in the relative importance of the evaluation criteria, enabling decision-makers to realise the value trade-offs they are willing to make and therefore construct and analyse their own value preferences. Thirdly, the methodological process can be informed through extensive expert engagement and direct stakeholder participation leading to an encompassing capture of value perceptions and value preferences. Fourthly, it provides flexibility given that the details and technical characteristics of the different methodological stages can be adapted to accommodate particular decision-makers’ needs. Finally, the entire process is fully transparent, allowing to illustrate the rationale behind the decision outcomes which could enable them to become more credible and well-accepted from the wider stakeholder community and society.

The resulting overall WPV scores derived from the MCDA process can act as a more encompassing measure of value given that multiple benefit dimensions are explicitly assessed and therefore could be used to drive the coverage decision and pricing negotiations of new medicines and health care interventions in a more comprehensive manner. Consideration of purchasing costs in parallel with the overall value of the alternatives options can then be used to estimate the incremental cost value ratio (ICVR) of the different health care interventions. Dividing costs by benefits reflect the associated opportunity cost, helping to obtain the best value-for-money alternatives and contribute to efficient priority setting and resource allocation [44]. For example, assuming the existence of a defined budget constraint for funding a set of alternative treatments, the most efficient options could be allocated based on their ICVR rankings, from the lowest (cost-benefit) ratio to the highest ratio, until the available budget is exhausted [66]. Incorporation of budget impact considerations in the cost-value ratio estimates at system level could take place by taking into account the number of patients that will receive the treatment, however benefits and costs should be estimated in comparable units so that the results are not biased towards the cheapest alternative, as for example on a per-patient basis [13].

In this context, the case study conducted aimed to assess and rank alternative treatment options for the same disease indication. A number of disease-specific clinical endpoints were incorporated as evaluation criteria which reflected a number of common value concerns relating to the particular intra-indication decision context of interest. Given the early and experimental stage of this methodology, further testing applications could target relatively expensive health care interventions for disease indications with high unmet need, or long term treatments for chronic conditions where multiple alternative options exist, essentially investigating coverage decisions where multiple benefits and/or high budget impact might be at stake.

Such an attempt at a broader inter-indication level, aiming to assess the value of alternative treatments across different disease indications might be more challenging as it would need to ensure the use of a common value model (in terms of attributes, value functions and relative weights) that adequately addresses the value concerns for the alternative treatments across diseases with different characteristics. In such an evaluation context, criteria and attributes might need to become more generic and less disease-specific, using health benefit metrics such as the QALY, which allow for the comparison of health gain across different patient populations. Therefore, analytical trade-offs might have to take place between the potential sensitivity in “picking-up” value through disease-specific health outcomes and the practicality of comparing value across patients through common criteria of generic health outcomes.

Another challenge would relate to designing an efficiency frontier or threshold (i.e. cut-off point) across diseases, essentially an alternative to the current ICER threshold. This issue would not be limited to the application of MCDA and would face all the theoretical and practical hurdles associated with the estimation of a sound cost-benefit threshold based on opportunity cost that have been seen to date [67, 68].

The preference elicitation process could be adjusted so that value preferences are not restricted to the appraisal committee or evaluation board responsible for decision-making. Instead, evidence on the preferences of the wider stakeholder community could be potentially incorporated in the evaluation, as for example by conducting discrete choice experiment studies to identify relative criteria importance in the form of weights, as in the case of societal preferences of citizens in Belgium [69].

## Conclusion

The challenge to assess novel treatments and therapeutic combinations in a setting of significant budgetary pressure on health services require novel methodologies of assessment allowing the incorporation of preferences from groups of stakeholders across a set of multiple value dimensions. In this study we described an integrated multi-criteria approach simulating an HTA context for the case of advanced colorectal cancer treatments. Innovative approaches to decision-making for pricing and reimbursement of new therapies will be essential in the coming era of precision medicine and expensive but effective immunotherapies for cancer. Ultimately, because of their characteristics enabling a structured process, MCDA methodologies such as the Advance Value Framework could overall facilitate HTA decision-making acting as a reasonable resource allocation tool that, among others, incorporates a more holistic and transparent approach to value assessment and value communication. Future research could test the Advance Value Framework methodology by conducting similar case studies with multi-stakeholder groups in different countries.

## Appendix

### Methodological process

At first, as part of the problem structuring phase, the decision problem and the aims of the analysis are defined, and the relevant decision-makers and other key stakeholders are identified. Next, as part of the model-building phase, objectives and/or relevant criteria are identified in order to reflect decision makers’ goals and areas of concern, and attributes are selected to operationalise the criteria. In addition, under the same phase, selection of the alternative options takes place and evidence on their performance across the selected criteria is identified. Following that, under the model assessment phase, the performance of options against the criteria is assessed (i.e. scoring) and criteria are weighted according to their relative importance (i.e. weighting). Subsequently, as part of the appraisal phase, scores and weights are combined in order to produce overall WPV scores, taking the form of a value index (i.e. aggregation). In combination with sensitivity analysis, the results are examined and their robustness is determined. Finally, as part of action planning, the outcome of the analysis can be used to inform resource allocation decisions, of a coverage or pricing nature.

### Clinical practice and scope of the exercise (problem structuring)

The TA242 scoping evaluated bevacizumab in combination with non-oxaliplatin chemotherapy, cetuximab monotherapy or combination with chemotherapy, and panitumumab monotherapy for mCRC after first-line chemotherapy. The populations covered for the case of cetuximab and panitumumab were mCRC patients expressing the wild-type (i.e. non-mutated) form of the v-Ki-ras2 Kirsten rat sarcoma viral oncogene homolog (KRAS) gene, because these agents, which target the epidermal growth factor receptor (EGFR), have been shown to be ineffective for treatment of tumours expressing the mutated KRAS gene [70–72]. KRAS expression does not impact on the case of bevacizumab. Bevacizumab was appraised only when in combination with non-oxaliplatin based chemotherapy, because under UK clinical practice oxaliplatin containing combinations (i.e. FOLFOX) are generally used in the first line. Once cancers demonstrate resistance to FOLFOX patients are then eligible for non-oxaliplatin based chemotherapy regimens such as FOLFIRI (irinotecan, 5-fluorouracil and folinic acid), therefore, patients treated with bevacizumab at second-line would normally receive it in combination with non-oxaliplatin based chemotherapies. Hence, the exclusion of oxaliplatin based chemotherapies from the scope of the TA and our exercise.

The scope of TA307 specified the evaluation of aflibercept in combination with FOLFIRI that has progressed following prior oxaliplatin based chemotherapy. Again, the scope of the TA and our exercise considered aflibercept only in combination with non-oxaliplatin chemotherapy for the same reason explained above.

### Evidence considered and alternative treatments compared (model building)

As part of TA242, for the case of cetuximab and panitumumab, NICE considered clinical evidence coming from two open label, Phase 3 RCTs respectively; the first one investigating the use of cetuximab plus best supportive care (BSC) compared to BSC alone (*CO.17* trial)^2^ [73] and the second one investigating the use of panitumumab plus BSC compared to BSC alone (*AMGEN* trial) [65], in patients with chemotherapy-refractory mCRC. For the case of bevacizumab, as part of TA242, only one RCT had been identified investigating bevacizumab as a second line treatment (*E3200* trial) [74]. However in that trial, bevacizumab was administered in combination with an oxaliplatin-containing chemotherapy which was outside the appraisal’s scope, and hence outside the scope of our analysis.

As part of TA307, the clinical evidence for aflibercept was taken from a prospective multinational, randomized, double-blind, parallel-arm, phase 3 study investigating the addition of aflibercept to FOLFIRI in patients with mCRC previously treated with an oxaliplatin-based regimen (*VELOUR* trial) [32].

Finally, for the case of regorafenib, no clinical evidence was considered as part of TA334 because no evidence submission was received from the manufacturer and the appraisal was terminated early.

No indirect comparison was conducted given the lack of a common comparator among the three treatments of interest in the above clinical studies. A mixed-treatment comparison lied outside the aim of the simulation exercise which was to operationalise the new value framework through the elicitation of preferences across a range of explicit criteria from a group of stakeholders. As a result, for the case of cetuximab and panitumumab clinical evidence was used from a latest head to head, open label, randomised, multicentre Phase 3 non-inferiority study directly comparing both treatments (*ASPECCT* trial) [33], whereas for the case of aflibercept in combination with non-oxaliplatin based chemotherapy evidence was used from the same clinical study that NICE considered. However, data from the BSC comparator arms of the two trials that NICE considered as part of TA242 for the case of cetuximab and panitumumab (*CO.17*, *AMGEN*) [65, 73] were used for the purpose of setting the reference levels on the attributes.

### Setting attribute ranges and reference levels (model building)

For the case of clinical therapeutic attributes, the “higher reference” levels were normally based on BSC figures, coming from the median of the respective arms of the *CO.17* and *AMGEN* trials; otherwise, if no BSC figure was available the placebo comparator arm from the *VELOUR* trial was used. The “lower reference” levels were based on the worst performances plausible, inferred either based on their lowest natural limit (for the case of continuous scale attributes, e.g. 0 months for OS) or based on the lowest evidence-based limit (for the case of non-natural constructed scale attributes, e.g. 0.6 utility for HRQoL as it was the lower utility used for progressive disease by NICE). The maximum levels of the attributes were simply derived by adding a 10% absolute increment to the performance level of the best performing option, essentially offering an error margin to the limits of the scale. This was performed to produce reference levels that corresponded to “worst performance” (plausible) and “satisfactory performance” (proxied by BSC), corresponding to the 0 and 100 anchor levels of the value function scale respectively, with options performing better than the satisfactory level scoring more than 100. By this way three attribute levels were defined in total: i) the “lower reference” level (x_l) (i.e. worst performance plausible), acting on the same time as minimum level (x_*); ii) the “higher reference” level (x_h) (i.e. BSC-based satisfactory performance); and iii) the maximum level (x^*) (i.e. 10% higher than the best performing option), to give x_*≤x_l < x_h< x^*.

For the purpose of eliciting preferences and producing the matrix of judgements using M-MACBETH, we aimed to incorporate two additional intermediate attribute levels lying in-between the three defined attribute levels (giving a total of five different attribute levels) so that the granularity of the scale is increased, essentially to improve the representation of any differences in value across the attribute ranges. In cases where the gaps between the three defined levels were disproportionate large, a third intermediate level was added for a more homogeneous dispersion, giving a total of six attribute levels (three defined and three intermediates), whereas in cases of disproportionate small gaps only one intermediate level was added giving a total of four attribute levels (three defined and one intermediate). In no cases there were less than four and more than six attribute levels in total.

Similar but reverse logic was adopted for setting the reference levels of the safety attributes; the “higher reference” levels were based either on the median of the BSC arm from the *AMGEN* trial and the placebo comparator arm of the *VELOUR* trial (BSC data from the *CO.17* trial were not available for all attributes), or if this was not relevant on the median of the options (e.g. for the case of the existence of contra-indications). The “lower reference” levels were derived either by adding a 10% absolute increment to the worst performing option (e.g. 10% higher incidence for the case of AEs) or by choosing the worst performance plausible for the case of a constructed attribute with a non-continuous scale (i.e. for the existence of contra-indications). The maximum level of the attributes was defined by selecting the best performance plausible (e.g. 0% for incidence of AEs), whereas the minimum level (i.e. worst performance) was equal to the “lower reference” level.

For the innovation attributes, the “higher reference” level was derived by using the median of the options (BSC performance was irrelevant to be used as satisfactory level), whereas the “lower reference” level was based on the worst performance plausible as inferred from the lowest limit of the scales (e.g. 5th entrance at an ATC level, or 0 number of new indications for which the technology is investigated in a given clinical development stage). The maximum level of the attributes was derived by either adding a 10% absolute increment to the performance level of the best performing option, for the case of natural attributes with a continuous scale (e.g. number of new indications for which the technology is investigated in a given clinical development stage), or alternatively by using the best performance plausible for the case of constructed attributes with discrete-level scales (e.g. 1^st^ entrance at an ATC level). The minimum level was equal to the “lower reference” level.

For the socioeconomics attribute (impact on direct costs), the “higher reference” level was derived by using the median of the options (BSC performance was irrelevant to be used as satisfactory level), and the “lower reference” level was derived by adding a 10% absolute increment to the worst performing option (i.e. to the one with the biggest impact on costs. The maximum level was defined by selecting the best performance plausible, as inferred from the highest natural limit of the scale (i.e. £0 impact on costs), whereas the minimum level (i.e. worst performance) was equal to the “lower reference” level.

### Decision conference (model assessment and appraisal)

Participants were contacted through an email invitation outlining the exercise and the purpose of the project, and background material introducing the scope of the exercise in more detail was sent one week before the workshop.

In terms of the decision-aiding methodology used, one of the authors (GM) acted as an impartial facilitator with the aim of enhancing content and process interaction, while refraining from contributing to the content of the group’s discussions, essentially guiding the group in how to think about the issues but not what to think [44, 75]. In terms of facilities, the room of the workshop had a Π-shaped meeting table for all the participants to have direct eye to eye contact, with an overhead projector screen surrounded by whiteboards. The M-MACBETH software was operated by one of the authors (AA) using a laptop, the screen of which was connected to the projector.

The workshop lasted the whole day, from 9.00 am to 18.00 pm with one 45-minutes lunch break, and two 15-minutes coffee breaks. The day started with a brief introduction by one of the authors (PK) and then moved on with an overview of the MCDA methodology adopted and the description of the value tree. The value tree was then presented and analysed cluster by cluster.

At the beginning of each cluster the value tree was validated; the various criteria were explained, followed by a group discussion relating to their relevance and completeness. As a result of this iterative process, some of the criteria were excluded because they were perceived as irrelevant or non-fundamental, but no criteria were deemed to be missing. Then, value functions were elicited for the different criteria and criteria weights were elicited within the clusters. Finally, relative weights were assigned across clusters, which enabled calculating the overall WPV scores of the options.

### MCDA technique (model assessment and appraisal)

MACBETH uses seven semantic categories ranging between “no difference” to “extreme difference”, in order to distinguish between the value of different attribute levels. Based on these qualitative judgements of difference and, by analysing judgmental inconsistencies, it facilitates the move from ordinal preference modeling, a cognitively less demanding elicitation of preferences, to a quantitative value function. An example of the type of questioning being asked would be “What do you judge to be the difference of value between x’ and “x”?” where x’ and “x” are two different attribute levels of attribute x, across the plausible range (i.e. x_*_ ≤ x’, “x” ≤ x^*^). The approach has evolved through the course of theoretical research and real world practical applications, making it an interactive decision support system that facilitates decision makers’ communication.

Following the elicitation of value functions, criteria baseline weights can be elicited. Questions of direct importance for a criterion such as “*How important is a given criterion*?” are known to be as one of the most common mistakes when making value trade-offs because they are assessing them independent of the respective attribute ranges [76]. In contrast, indirect weighting techniques that assess value trade-offs in tandem with the respective ranges of attributes should be employed. For example, the quantitative swing weighting technique asks for judgments of relative value between “swings” (i.e. changes, from standard lower level x_l to higher reference level x_h on each x-th attribute) taking the form “*How would you rank the relative importance of the criteria considering their attribute ranges, relative to 100 for the highest-ranked criterion*?”. Each swing, i.e. a relative change from a lower attribute level to a higher attribute level, is valued between 0 and 100, with the most valuable swing anchored as 100 [24]. Normalised weights are then calculated, as a proportion of each swing weight, so the normalised weights are summed to 100%. Instead, relative attribute weights were calculated using an alternative qualitative swing weighting protocol, by using the MACBETH procedure to elicit the differences in attractiveness between the lower and higher reference levels of the different attributes, initially at individual level and then at criteria cluster level (i.e. by considering multiple attribute swings on the same time) [48, 49].

Finally criteria preference value scores and the respective weights can be combined together through an additive aggregation approach as described in Eq. 2 (if the conditions of complete and transitive preferences are met as well as multi-attribute preferential independence conditions) [24].

### Options performance, criteria weights and overall preference value rankings

Example of value judgements matrices for the Overall Survival and Health Related Quality of Life attributes and their conversion into value functions using the M-MACBETH software:

* Images produced using the M-MACBETH (beta) software version 3.0.0

Caption: In this example, the question asked for the case of OS was the following: “*What do you judge to be the difference of value between 0 months OS and 3 months OS? No difference, very weak, weak, moderate, strong, very strong, or extreme?*” Once a consensus was reached, the next question came along: “*What do you judge to be the difference of value between 0 months OS and 6.2 months OS? No difference, very weak, weak, moderate, strong, very strong, or extreme?”* The same process was followed until value judgments for all the different combinations of attribute levels were elicited, filling in the different rows from the right-hand side (i.e. lower range) to the left-hand side (i.e. higher range), bottom to top.

### Sensitivity and robustness analysis

Sensitivity analysis on weights for Cetuximab versus Panitumumab:

Sensitivity analysis on weights for Panitumumab vs Aflibercept plus FOLFIRI:

Sensitivity analysis on weights for Cetuximab vs Aflibercept plus FOLFIRI:

Robustness analysis on reference levels:

Caption: Red triangles denote “dominance” (an option dominates another if it is at least as attractive as the other in all criteria and it is more attractive than the other in at least one criterion). Green crosses denote “additive dominance” (an option additively dominates another if it is always found to be more attractive than the other through the use of an additive model under a set of information constraints).

For more information please see M-MACBETH user manual [53].

## Abbreviations

AEs: Adverse events
ATC: Anatomical Therapeutic Chemical
AVF: Advance Value Framework
BoD: Burden of disease
BSC: Best supportive care
DA: Decision analysis
ERG: Evidence Review Group
HRQoL: Health Related Quality of Life
HTA: Health technology assessment
ICER: Incremental cost effectiveness ratio
ICVR: Incremental cost value ratio
INN: Innovation level
LSE: London School of Economics and Political Science
MAVT: Multi-Attribute Value Theory
MCDA: Multiple criteria decision analysis
mCRC: Metastatic colorectal cancer
NICE: National Institute for Health and Care Excellence
ORR: Objective response rate
OS: Overall survival
RCTs: Randomised clinical trials
SAF: Safety profile
SOC: Socioeconomic impact
SPCs: Summaries of product characteristics
TA: Technology Appraisal
THE: Therapeutic impact
WPV: Weighted preference value

## Footnotes

1 Advance-HTA was a research project funded by the European Commission’s Research Framework Programme (FP7). It comprised several complementary streams of research that aimed to advance and strengthen the methodological tools and practices relating to the application and implementation of Health Technology Assessment (HTA). It was a partnership of 13 Consortium members led by the London School of Economics - LSE Health.
